# Clinical application of the F21 multipurpose cystoscope with continuous irrigation capability in photoselective vaporization of the prostate: a retrospective controlled study

**DOI:** 10.3389/fsurg.2026.1848723

**Published:** 2026-06-08

**Authors:** Yin Yu, Tao Ma, Lijun Zhou, Jianjun Guo, Guihua Cao, Zhusheng Zhu, Qiang Li, Ling Wang

**Affiliations:** 1 Department of Urology, The People’s Hospital of Leshan, Leshan, China; 2 Department of Medical Psychology, Sichuan Provincial Corps Hospital, Chinese People’s Armed Police Forces, Leshan, China

**Keywords:** benign prostatic hyperplasia, continuous irrigation, cystoscope, photoselective vaporization of the prostate, sheath caliber, urethral stricture

## Abstract

**Objective:**

To evaluate whether an F21 multipurpose cystoscope with continuous irrigation capability is associated with a lower incidence of postoperative urethral stricture after photoselective vaporization of the prostate (PVP), while maintaining satisfactory short-term functional outcomes.

**Methods:**

This single-center retrospective comparative cohort included consecutive male patients aged ≥60 years who underwent PVP between January 2024 and January 2025. Procedures were performed using either the F21 multipurpose cystoscope with continuous irrigation capability or a conventional F26 resectoscope. All patients underwent standardized postoperative follow-up at 3 and 6 months, including assessment of the International Prostate Symptom Score (IPSS), maximum urinary flow rate (Qmax), postvoid residual urine volume (PVR), and clinical symptoms suggestive of urethral stricture. Postoperative urethroscopy was not performed routinely in all asymptomatic patients; instead, diagnostic urethroscopy was performed in patients with new or persistent obstructive voiding symptoms, a marked decrease in Qmax, increased PVR, or clinical suspicion of urethral stricture. Urethral stricture was defined as obstructive voiding symptoms combined with endoscopic confirmation of urethral narrowing with a caliber of <15 Fr. Between-group comparisons and multivariable logistic regression were used to assess the association between instrument type and urethral stricture while accounting for clinically relevant covariates.

**Results:**

A total of 240 patients were included, of whom 50 underwent PVP with the F21 instrument and 190 with the F26 resectoscope. Baseline characteristics and key perioperative variables were comparable between groups. Both groups showed marked postoperative improvement in IPSS and Qmax. Compared with the F26 group, the F21 group had significantly lower PVR at 3 months (2.316 ± 11.463 vs. 6.559 ± 16.714 mL, *p* = 0.038) and 6 months (0.520 ± 3.677 vs. 4.458 ± 14.020 mL, *p* = 0.001). The incidence of postoperative urethral stricture was lower in the F21 group than in the F26 group [2/50 (4.0%) vs. 28/190 (14.7%), *p* = 0.041].

**Conclusion:**

In this retrospective cohort study, the use of the F21 multipurpose cystoscope with continuous irrigation capability during PVP was associated with a lower rate of clinically detected postoperative urethral stricture and improved postoperative bladder emptying, while maintaining short-term improvements in urinary symptoms and urinary flow. However, given the retrospective design, symptom-triggered urethroscopic confirmation, and limited 6-month follow-up period, these findings should be interpreted as short-term, hypothesis-generating clinical evidence. Because urethral strictures may occur beyond 6 months after surgery, further prospective studies with longer follow-up periods, ideally at least 12–24 months, are warranted to validate these results.

## 1 Introduction

Benign prostatic hyperplasia (BPH) is one of the most common urological diseases among middle-aged and elderly men worldwide, and its incidence increases progressively with age (1, 2). Epidemiological studies have shown that approximately 60% of men develop BPH by the age of 65 years (3). At present, transurethral plasmakinetic resection, laser enucleation, and photoselective vaporization of the prostate (PVP) are the main surgical treatment options for BPH (4–6). However, urethral stricture remains a clinically relevant complication after transurethral prostate surgery. Previous studies have reported that the incidence of postoperative urethral stricture ranges from 4.5% to 13%, with the membranous urethra being the most frequently involved site (7). To date, evidence on effective strategies for reducing the risk of urethral stricture after BPH surgery remains limited. Some studies have suggested that smaller-caliber transurethral instruments may help reduce this risk, whereas others have found no significant difference in postoperative urethral stricture rates between 24 Fr and 28 Fr sheaths (8, 9).

**Figure 1:**
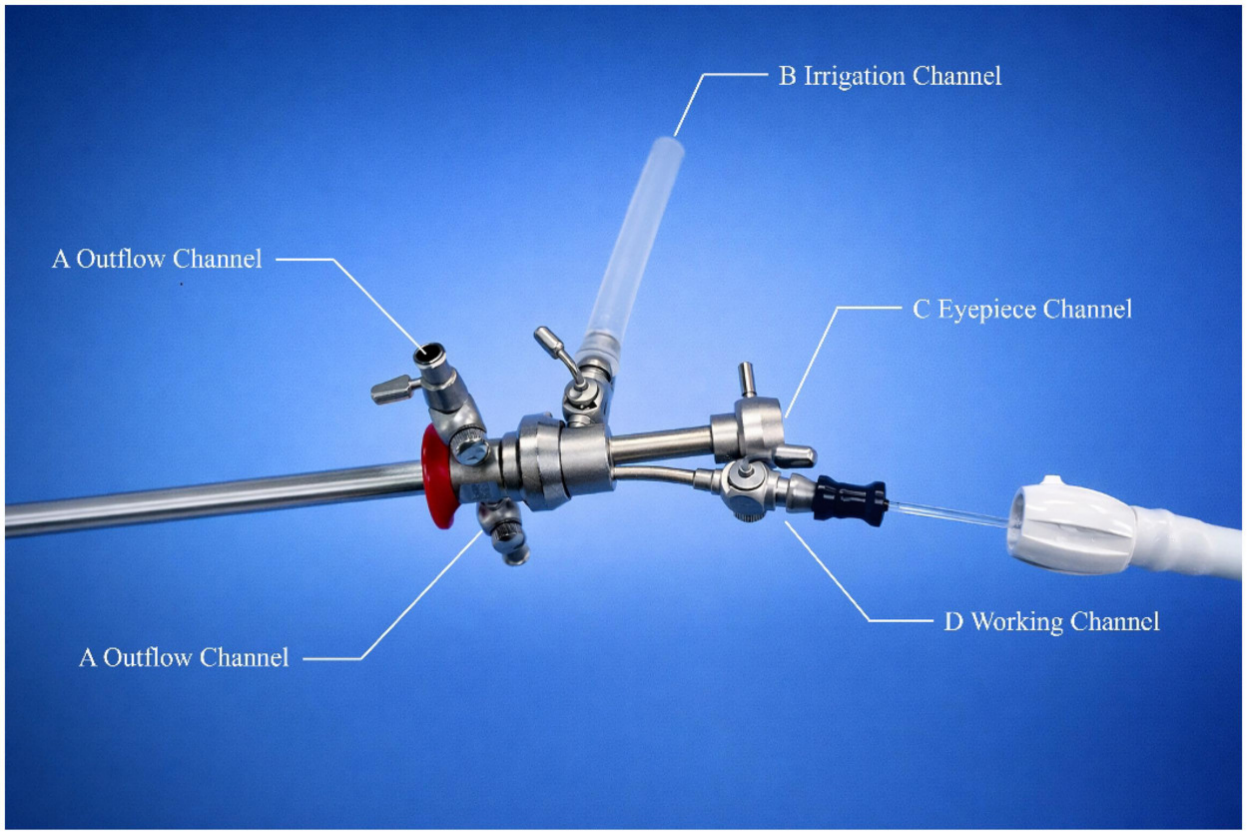
Gross appearance and structural features of the F21 multipurpose cystoscope with continuous irrigation capability.

Based on the hypothesis that a smaller-caliber instrument may reduce postoperative urethral stricture by minimizing intraoperative mechanical injury to the urethral mucosa, we designed and developed an F21 multipurpose cystoscope with continuous irrigation capability (utility model patent no. 202323218027.1; Supplementary File 1; Figure 1). We then conducted a retrospective comparative study against the conventional F26 resectoscope to evaluate its potential advantage in reducing the incidence of urethral stricture after PVP. According to the EAU grading criteria for male urethral stricture (10), urethral stricture in the present study was defined as endoscopically confirmed narrowing of the urethral lumen (urethral caliber <15 Fr) accompanied by corresponding obstructive voiding symptoms.

## 2 Study design

This study was designed as a single-center retrospective comparative cohort study of patients who underwent PVP for benign prostatic hyperplasia between January 2024 and January 2025. During the study period, 467 patients underwent PVP in our department. Consecutive cases were screened according to predefined inclusion and exclusion criteria, and 240 eligible patients were included in the final analysis. The choice of the F21 multipurpose cystoscope or the conventional F26 resectoscope was based on instrument availability and routine clinical practice rather than random allocation; therefore, potential selection bias and confounding were considered in the study design and statistical analysis.

To reduce procedural heterogeneity, all operations were performed by the same senior chief surgeon with more than 15 years of experience in PVP, and both groups followed the same perioperative protocol, including anesthesia, patient position, laser power settings, operative steps, irrigation strategy, catheter type, and postoperative catheter removal schedule. Baseline demographic characteristics, prostate-related variables, perioperative variables, and functional outcomes were compared between groups.

Given the retrospective and observational nature of the study, registration in a public clinical trial registry was not required. The study protocol was approved by the Ethics Committee of The People's Hospital of Leshan [Approval No. LYLL (2024) KY 137] and was conducted in accordance with the Declaration of Helsinki. Written informed consent was obtained from all participants prior to enrollment.

## 3 Clinical data

### 3.1 Inclusion criteria

Patients were eligible for inclusion if they met all of the following criteria: age ≥60 years, a preoperative International Prostate Symptom Score (IPSS) of 15–30, a prostate-specific antigen (PSA) level <4 ng/mL, a prostate volume of 40–80 mL confirmed by transrectal ultrasonography, and treatment with transurethral PVP performed using either an F26 resectoscope or an F21 multipurpose cystoscope.

### 3.2 Exclusion criteria

Patients were excluded if they met any of the following criteria: (1) a previous diagnosis of prostate cancer, bladder cancer, or other malignancies; (2) a history of diabetes mellitus, neurogenic bladder, or Parkinson’s disease; (3) prior urethral injury or urethral surgery; (4) a confirmed diagnosis of urethral stricture before or during surgery; or (5) missing key clinical data required for the present statistical analysis.

## 4 Methods

### 4.1 Patients who underwent PVP using a conventional F26 resectoscope

All procedures were performed by the same senior chief surgeon, who had more than 15 years of experience in PVP. Under combined intravenous–inhalation anesthesia, patients were placed in the lithotomy position, followed by routine perineal disinfection and sterile draping. A conventional F26 resectoscope was inserted transurethrally to sequentially inspect the urethra, verumontanum, prostate, bladder, and bilateral ureteral orifices. The GreenLight laser system was then connected, and the laser fiber was introduced. The vaporization and coagulation power settings were 80 and 40 W, respectively.

Under direct vision, with the verumontanum serving as the distal safety landmark, the scope was maintained in the midline and continuous saline irrigation was used throughout the procedure. Initial vaporization incisions were made at the 5 and 7 o’clock positions to establish drainage channels, followed by stepwise layered vaporization of the lateral and median lobes to enlarge the prostatic urethral lumen until whitish fibrous tissue resembling the surgical capsule was exposed. Particular care was taken to preserve the distal external urethral sphincter beyond the verumontanum, as well as the bladder neck. Hemostasis was performed simultaneously during the procedure, and coagulation mode was applied when necessary to control bleeding points and maintain a clear operative field.

At the end of the procedure, the urethra was re-examined, and no obvious urethral injury was identified. A 20-Fr three-way latex urinary catheter was then inserted transurethrally, and the balloon was inflated with 20 mL of sterile water. The catheter was secured under traction, and continuous bladder irrigation was established. Continuous bladder irrigation was discontinued 24 h after surgery, and the catheter was removed 1 week postoperatively.

### 4.2 Patients who underwent PVP using the F21 multipurpose cystoscope

**Figure 2:**
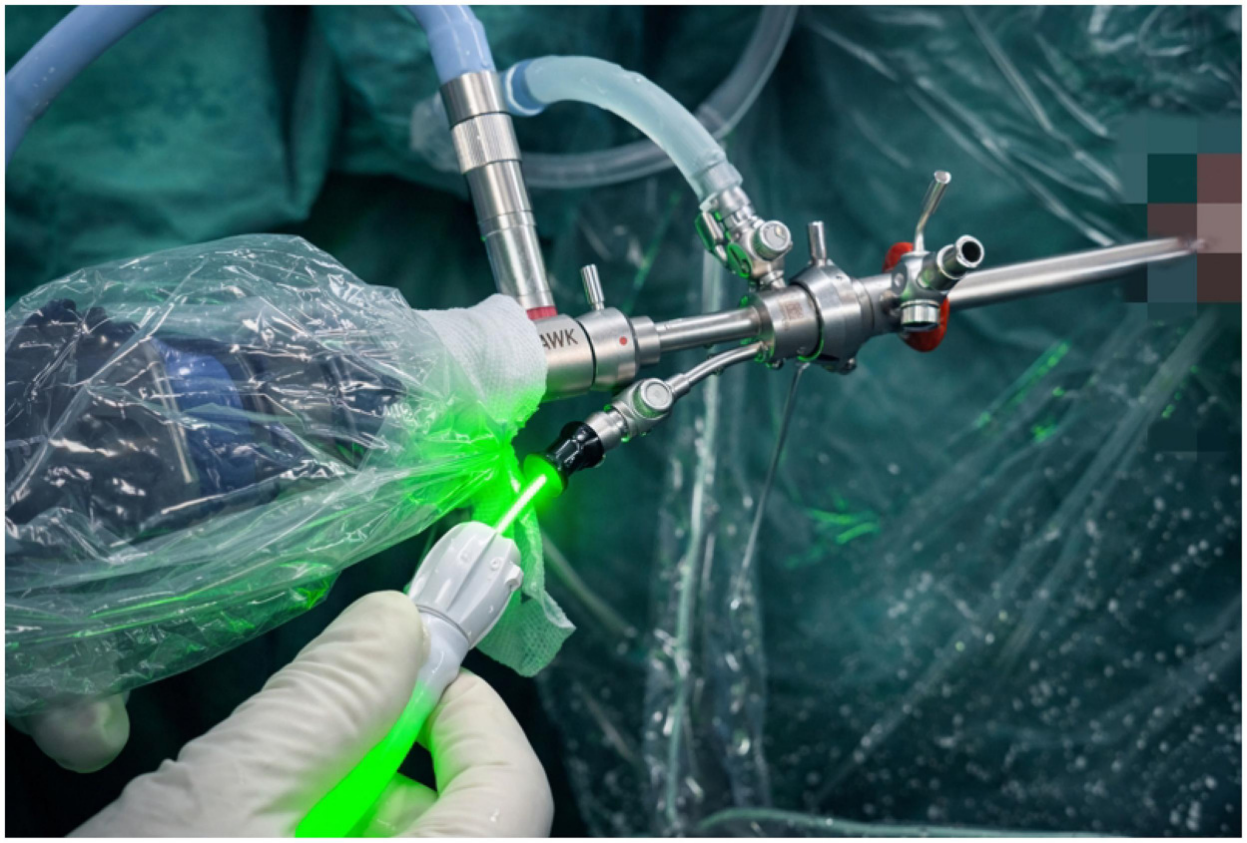
Intraoperative application of the F21 multipurpose cystoscope during PVP.

The only procedural difference was the use of the F21 multipurpose cystoscope instead of the conventional F26 resectoscope; all other operative steps were identical to those performed in the F26 group (Figure 2).

### 4.3 Data collection

All patients were scheduled for standardized follow-up visits at 3 and 6 months after surgery. At each follow-up visit, urinary symptoms were assessed using IPSS, and objective voiding parameters were evaluated using Qmax and PVR. Patients were also specifically asked about obstructive symptoms potentially suggestive of urethral stricture, including a weak urinary stream, spraying, straining, prolonged voiding time, incomplete emptying, urinary retention, or recurrent difficulty in catheterization.

Postoperative urethroscopy was not performed routinely in all asymptomatic patients because of the retrospective clinical-practice setting and the invasive nature of the procedure. Diagnostic urethroscopy was performed in patients with new or persistent obstructive voiding symptoms, a marked reduction in Qmax, increased PVR, or clinical suspicion of urethral stricture during follow-up. Urethral stricture was defined as the coexistence of obstructive voiding symptoms and endoscopic confirmation of urethral narrowing with a caliber of <15 Fr, in accordance with the diagnostic criterion used in this study.

This symptom-triggered diagnostic strategy reflects routine clinical practice but may miss asymptomatic or minimally symptomatic strictures. Therefore, the reported stricture rate should be interpreted as the incidence of clinically detected postoperative urethral stricture within 6 months rather than the absolute incidence of all anatomical urethral narrowing. The present study focused on clinically detected urethral stricture within 6 months after PVP. We acknowledge that late-onset urethral strictures may occur beyond this follow-up window; therefore, the reported incidence may underestimate the true long-term incidence of urethral stricture.

### 4.4 Statistical analysis

All data were organized and analyzed using SPSS version 27.0. Continuous variables are presented as mean ± standard deviation (SD). Normality was evaluated before between-group comparison. Normally distributed continuous variables were compared using the independent-samples t test, whereas non-normally distributed variables were compared using the Mann–Whitney U test when appropriate. Categorical variables are expressed as number and percentage [n (%)] and were compared using the *χ*^2^ test or Fisher's exact test, as appropriate.

All statistical tests were two-sided, and a *p* value <0.05 was considered statistically significant. The sample size was statistically estimated during the study design phase. Based on previously published relevant studies and preliminary results, the effect size of the primary outcome was defined, and the sample size was calculated using a two-sided test with a significance level of *α* = 0.05 and a statistical power of 0.80, for example using G*Power software. The results indicated that a minimum sample size of 30 patients in each group would be sufficient to meet the requirements for statistical testing. In the present study, the final numbers of patients included in both the F21 multipurpose cystoscope group (*n* = 50) and the F26 resectoscope group (*n* = 190) exceeded the minimum required sample size.

## 5 Results

Between January 2024 and January 2025, a total of 467 patients underwent PVP in our department. After screening according to the predefined inclusion and exclusion criteria, 240 patients were finally enrolled, including 50 in the F21 multipurpose cystoscope group and 190 in the F26 resectoscope group. To assess the baseline comparability between the two groups, independent-samples *t* tests were performed for key clinical and perioperative variables, including age, body mass index (BMI), PSA, prostate volume(PV), operative time(OT), irrigation duration, and length of hospital stay (Table 1).

**Table 1:** Baseline characteristics and perioperative variables of the study population.

Outcome	Group (mean ± SD)		*t*	*p*
	F21(*n* = 50)	F26(*n* = 190)		
Age	71.520 ± 8.170	71.711 ± 7.947	−0.150	0.881
BMI	25.654 ± 0.836	25.459 ± 0.858	1.431	0.154
PSA	1.795 ± 1.241	1.873 ± 1.214	−0.403	0.687
PV	53.480 ± 16.022	53.147 ± 16.186	0.130	0.897
Operative time	65.100 ± 16.867	64.442 ± 16.377	0.251	0.802
Irrigation time	16.900 ± 4.404	17.026 ± 4.277	−0.185	0.854
Hospital stay	5.380 ± 1.689	5.042 ± 1.436	1.425	0.155

Baseline demographic characteristics and perioperative variables were comparable between the two groups, with no statistically significant differences observed in age, BMI, PSA, prostate volume, operative time, irrigation time, or hospital stay (all *p* > 0.05). Overall, the F21 and F26 groups were well balanced with respect to baseline clinical characteristics and key perioperative variables, supporting good comparability between groups and reducing the potential influence of confounding factors on subsequent analyses of clinical outcomes.

Independent-samples t tests were used to compare key voiding function parameters, including the International Prostate Symptom Score (IPSS), maximum urinary flow rate (Qmax), postvoid residual urine volume (PVR), between the F21 and F26 groups at baseline and at 3 and 6 months after surgery, in order to evaluate differences in clinical efficacy between the two interventions (Table 2).

**Table 2:** Comparison of baseline and postoperative follow-up outcomes between the two groups.

Outcome	Group (mean ± SD)		*t*	*p*
	F21	F26		
Preoperative IPSS	26.940 ± 4.235	27.295 ± 4.553	−0.497	0.620
IPSS at 3 months	1.500 ± 3.358	2.053 ± 4.486	−0.960	0.339
IPSS at 6 months	1.120 ± 3.515	2.274 ± 4.974	−1.878	0.063
Preoperative Qmax	3.240 ± 1.975	3.011 ± 1.935	0.743	0.458
Qmax at 3 months	21.800 ± 5.163	20.674 ± 8.093	1.202	0.232
Qmax at 6 months	22.960 ± 5.075	21.284 ± 8.392	1.781	0.077
Preoperative PVR	53.940 ± 8.484	54.700 ± 6.064	−0.595	0.554
PVR at 3 months	2.316 ± 11.463	6.559 ± 16.714	−2.096	0.038
PVR at 6 months	0.520 ± 3.677	4.458 ± 14.020	−3.447	0.001

At baseline, all measured parameters were comparable between the two groups, with no statistically significant differences observed (all *p* > 0.05). Specifically, the preoperative IPSS was 26.940 ± 4.235 in the F21 group and 27.295 ± 4.553 in the F26 group (t = −0.497, *p* = 0.620), while the corresponding preoperative Qmax values were 3.240 ± 1.975 and 3.011 ± 1.935 mL/s, respectively (t = 0.743, *p* = 0.458). These findings indicate good baseline comparability between the two groups.

At 3 months after surgery, Qmax was 21.800 ± 5.163 mL/s in the F21 group and 20.674 ± 8.093 mL/s in the F26 group (*t* = 1.202, *p* = 0.232), whereas IPSS was 1.500 ± 3.358 and 2.053 ± 4.486, respectively (*t* = −0.960, *p* = 0.339). PVR was significantly lower in the F21 group than in the F26 group (2.316 ± 11.463 vs. 6.559 ± 16.714 mL, *t* = −2.096, *p* = 0.038). At 6 months after surgery, Qmax was 22.960 ± 5.075 mL/s in the F21 group and 21.284 ± 8.392 mL/s in the F26 group (*t* = 1.781, *p* = 0.077), and IPSS was 1.120 ± 3.515 and 2.274 ± 4.974, respectively (*t* = −1.878, *p* = 0.063). PVR remained significantly lower in the F21 group than in the F26 group (0.520 ± 3.677 vs. 4.458 ± 14.020 mL, *t* = −3.447, *p* = 0.001).

Overall, although postoperative Qmax and IPSS showed a trend toward better outcomes in the F21 group, these differences did not reach statistical significance (*p* > 0.05). In contrast, PVR at both 3 and 6 months was significantly lower in the F21 group, indicating that both interventions achieved satisfactory short-term improvement in voiding function and symptom relief, while the F21 approach showed a greater advantage in improving postoperative bladder emptying.

The incidence of postoperative urethral stricture was compared between the F21 and F26 groups using the *χ*^2^ test to evaluate differences in safety between the two interventions (Table 3). In the F21 group, 2 of 50 patients developed postoperative urethral stricture (4.0%), whereas 48 patients (96.0%) did not. In the F26 group, 28 of 190 patients developed urethral stricture (14.7%), while 162 patients (85.3%) remained free of this complication.

**Table 3:** Comparison of postoperative urethral stricture between the two groups.

Outcome		Group (%)		*χ* ^2^	*p*
		F21	F26		
Urethral stricture	No	48 (96.000)	162 (85.263)	4.172	0.041
	Yes	2 (4.000)	28 (14.737)		
Total		50	190		

Between-group comparison showed a statistically significant difference (*χ*^2^ = 4.172, *p* = 0.041), indicating that the incidence of postoperative urethral stricture was significantly lower in the F21 group than in the F26 group. These findings suggest that, while maintaining comparable clinical efficacy, the F21 approach may offer improved safety by reducing the risk of postoperative urethral stricture.

## 6 Discussion

According to the relevant section of the European Association of Urology (EAU) Guidelines, the reported incidence of urethral stricture after transurethral prostatic surgery is approximately 4.5%–13%, with the bulbomembranous urethra being the most commonly affected site (7). The guidelines further propose several potential mechanisms underlying stricture formation, including instrument-related friction, inadequate lubrication, repeated insertion and withdrawal of instruments, and consequent urethral mucosal injury.Against this background, the 4.0% stricture rate observed in the F21 group in the present study was comparable to the 3.4%–4.4% reported in previous PVP series, whereas the rate in the F26 group (14.7%) was noticeably higher. These findings suggest that, beyond the energy modality itself, sheath caliber may play an important role in the development of postoperative urethral stricture. Supporting this view, an observational study of transurethral resection of the prostate demonstrated that a larger sheath diameter may be associated with a higher risk of urethral stricture; compared with the use of a 26 Fr sheath, intraoperative use of a 24 Fr sheath was associated with a lower incidence of postoperative urethral stricture (11).

However, the effect of instrument caliber has not been entirely consistent across studies. Some reports have placed greater emphasis on patients’ anatomical characteristics, such as meatal caliber, rather than sheath size itself. In a prospective observational study of TURP, a smaller urethral meatal caliber was found to be significantly associated with an increased risk of urethral stricture, whereas sheath caliber (24 Fr vs. 26 Fr) showed no significant association with stricture formation (12).At a higher level of evidence, systematic reviews and meta-analyses provide external support for the present study's finding of preserved efficacy with an optimized safety profile. A meta-analysis including 69 randomized controlled trials and 8,517 patients demonstrated that, compared with TURP, PVP achieved comparable short-term efficacy while offering shorter hospitalization and fewer complications. In addition, a meta-analysis of randomized trials focusing on urethral stricture after different endoscopic surgical procedures showed that the incidence of urethral stricture was higher in the TURP group than in the enucleation/ablation group, and identified sheath caliber and postoperative catheterization duration as important factors associated with stricture development (8). Therefore, the findings of the present study support the notion that, within the same energy modality and surgical framework, reducing instrument-related injury may be an important pathway for decreasing postoperative complications. Against the background of still limited high-level evidence, we designed and applied the F21 multipurpose cystoscope with continuous irrigation capability based on the hypothesis that smaller-caliber instruments may reduce mechanical injury to the urethral mucosa, and compared it with the conventional F26 resectoscope in a single-center retrospective controlled study.

The most important finding of the present study was that, while maintaining comparable short-term efficacy, the F21 group exhibited a significantly lower incidence of postoperative urethral stricture than the F26 group. Specifically, urethral stricture occurred in 2 of 50 patients (4.0%) in the F21 group and in 28 of 190 patients (14.737%) in the F26 group, with a statistically significant difference between the two groups (*χ*^2^ = 4.172, *p* = 0.041). These findings suggest that reducing instrument caliber may represent a feasible strategy for lowering the risk of urethral stricture after PVP, particularly in elderly patients with BPH who require transurethral intervention.

With respect to functional outcomes, both groups achieved significant postoperative improvements in IPSS and Qmax at 3 and 6 months, without significant between-group differences, suggesting that the smaller-caliber instrument did not compromise the effectiveness of obstruction relief. Notably, the F21 group showed greater improvement in postvoid residual urine volume at both follow-up time points, indicating a potential advantage of the smaller-diameter instrument in improving voiding efficiency, facilitating bladder emptying, and reducing postoperative residual urine. Clinically, this may translate into a lower risk of urinary retention, fewer lower urinary tract symptoms, and an improved patient experience. These findings also indirectly support the potential benefits of the F21 device in terms of less tissue trauma and faster recovery.

From a mechanistic perspective, urethral stricture represents a fixed narrowing of the urethral lumen resulting from fibrotic remodeling of the urethral mucosa and the surrounding corpus spongiosum (10). The development of urethral stricture is generally associated with several factors, including mechanical injury to the urethral mucosa, local ischemia, inflammatory responses, and subsequent scar formation. In the setting of PVP, the F21 device may reduce the incidence of urethral stricture, compared with the F26 device, through several potential mechanisms: by decreasing contact pressure and friction during instrumentation, reducing microtrauma to the urethral mucosa and local ischemic injury, and thereby limiting subsequent scar formation. In addition, the F21 incorporates a continuous irrigation design, which may theoretically improve visualization and cooling during the procedure, reduce the need for repeated insertion and withdrawal to maintain a clear operative field, and indirectly lessen mucosal injury (13). This hypothesis is consistent with the original rationale of the present study, namely, that smaller-caliber instruments may reduce the incidence of urethral stricture by minimizing intraoperative mechanical injury to the urethral mucosa.

The present study provides a clear direction at both the theoretical and practical levels. Theoretically, it moves the discussion of reducing iatrogenic urethral stricture beyond surgical technique and energy modality alone, and advances the testable hypothesis that optimization of instrument design and caliber may play a critical role. Practically, without altering the key procedural steps of PVP, the use of a smaller-caliber endoscope with a continuous irrigation design may represent an effective strategy for reducing postoperative complications associated with prostate PVP. In the future, our center plans to conduct a multicenter prospective randomized controlled study with an extended follow-up of at least 12–24 months, while incorporating stricture site localization and objective assessments of urethral injury, such as intraoperative mucosal injury grading and postoperative imaging, to further clarify the causal relationship and determine the true clinical benefit.

Overall, the present study showed, within the short-term follow-up period, that compared with the conventional F26 resectoscope, the F21 multipurpose cystoscope with continuous irrigation capability used for PVP was able to further reduce postvoid residual urine while maintaining comparable improvements in IPSS and Qmax, and may also contribute to a lower incidence of postoperative urethral stricture. This finding is generally consistent with previous evidence and mechanistic hypotheses suggesting that sheath caliber and catheter-related factors may influence the development of urethral stricture. However, given the retrospective design, the lack of clarification regarding several key confounding variables, and the limited follow-up duration, the current evidence is more suitable for hypothesis generation and procedural optimization rather than for establishing a definitive causal relationship.

This study has several limitations. First, it was a single-center retrospective study, and the selection of the F21 multipurpose cystoscope or the conventional F26 resectoscope was not randomized. Instrument selection may have been influenced by instrument availability, clinical workflow, surgeon preference, or unmeasured anatomical characteristics. Although baseline characteristics were comparable and adjusted analyses were performed, residual confounding cannot be completely excluded. Second, the group sizes were unequal, with fewer patients in the F21 group than in the F26 group, which may reduce the precision of effect estimation. Third, the follow-up duration was limited to 6 months, whereas some urethral strictures may develop or become symptomatic at 12–24 months after surgery; therefore, the reported stricture rate should be interpreted as the incidence of clinically detected short-term urethral stricture rather than the true long-term incidence. Fourth, detailed stricture localization was not systematically available for all patients, limiting further anatomical analysis of whether the F21 device reduces injury at specific vulnerable sites such as the meatal, bulbar, or membranous urethra. Fifth, the present study mainly focused on urethral stricture and voiding function outcomes, while broader perioperative complications, including infection, bleeding, readmission, and reintervention, were not comprehensively evaluated. Future prospective multicenter randomized studies with longer follow-up, standardized stricture localization, and comprehensive complication reporting are warranted.

## 7 Conclusion

In this single-center retrospective cohort, use of the F21 multipurpose cystoscope with continuous irrigation capability during PVP was associated with a lower rate of clinically detected postoperative urethral stricture and lower postoperative PVR, without compromising short-term improvements in IPSS or Qmax. These findings support the potential value of optimizing endoscopic instrument design and sheath caliber in PVP. However, because of the retrospective design, non-randomized instrument selection, symptom-triggered urethroscopic confirmation, and limited 6-month follow-up, larger prospective multicenter studies with longer follow-up are required before definitive causal conclusions can be made.

## Data Availability

The datasets presented in this study can be found in online repositories. The names of the repository/repositories and accession number(s) can be found in the article/Supplementary Material.
